# Cochlear implantation with Slim Modiolar Electrode carriers enables hearing preservation

**DOI:** 10.1007/s00405-025-09267-2

**Published:** 2025-02-26

**Authors:** Kenan Dennis Sakmen, Christian Issing, Caterina Vazzana, Tobias Weißgerber, Albrecht Linke, Timo Stöver, Silke Helbig

**Affiliations:** 1 Department of Otorhinolaryngology, Head and Neck Surgery, Goethe University Frankfurt, University Hospital, Theodor-Stern-Kai 7, 60590 Frankfurt, Germany; 2 Department of Audiology and Audiological Acoustic, Goethe University Frankfurt, University Hospital, Theodor-Stern-Kai 7, 60590 Frankfurt, Germany

**Keywords:** Cochlear implant, Perimodiolar, Electrode carrier, Hearing preservation, Structure preservation, Slim Modiolar

## Abstract

**Purpose:**

To investigate hearing preservation in patients who received a cochlear implant with a preformed electrode carrier, namely the Slim Modiolar (SM).

**Methods:**

A monocentric, retrospective study was conducted. A total of 51 adult patients (24 females, 27 males), representing 60 ears, were included in the final evaluation.

The mean age was 60.9 ± 15.2 years (range 17.5–89.7 years). All patients had some degree of residual hearing. Frequency-specific pure-tone audiometry was performed at three time points: preoperatively (T0), within 30 days postoperatively (T1), and at a later time point during follow-up (T2; 20.7 ± 17.6 months). Low frequency pure tone average and the HEARRING group formula were used to facilitate comparison of hearing preservation rates with other results published so far.

**Results:**

In the first month after surgery, no significant deterioration was observed for the low frequencies, but a significant deterioration was observed at follow-up. Using the HEARRING classification system, the average short-term (T0–T1) hearing preservation score was 70%, which corresponds to partial hearing preservation. During follow-up (T0–T2), the score decreased to 61% in the study group, still representing partial hearing preservation. Complete hearing loss occurred in 20% of the cases.

**Conclusion:**

Especially in the low frequency range, the utilisation of SM electrode carriers during cochlear implantation facilitates preservation of residual hearing.

## Introduction and background

The positive effects of structure and function preserving cochlear implant (CI) surgery have been demonstrated many times over the past decades. In cases of substantial residual hearing after surgery the resulting combination of acoustic hearing and electrical stimulation in the same ear (electric-acoustic stimulation, EAS) [1] offers obvious advantages in terms of speech perception, especially under difficult listening conditions [2–4]. Since preservation of residual hearing is considered an indicator of structural preservation, it is important to minimise mechanical trauma to the delicate cochlear structures during insertion to achieve this goal. For this reason, CI surgeons have adapted their surgical approach to access the cochlea, for example by using the round window instead of a cochleostomy during cochlear implantation [5, 6]. This has helped to reduce bone dust and acoustic trauma during the drilling procedure [7].

In addition to surgical optimisation, electrode arrays have been improved over the years. For decades lateral wall (LW) electrode arrays, designed very thin and flexible, have been preferred for hearing preservation (HP) surgery. Adapting their diameter to the size of the round window and their improved flexibility has allowed intracochlear structure preservation during insertion [8, 9]. Despite these results supporting the use of LW electrode arrays [10], manufacturers focused also on the development of pre-shaped electrodes for use in HP surgery. These electrodes were designed to achieve a modiolus hugging position. Initial attempts were not very successful. The advance-off-stylet technique used with the Contour electrode that is available with the *CI24 RE(CA), CI512* or *CI612* implant (Cochlear Ltd., Macquarie, Australia) was reported to result in scalar deviations in several cases [11]. As a result, structure and therefore residual hearing was not preserved to the same extent as with straight electrode carriers [12].

In 2019, the Slim Modiolar (SM), a very thin, preformed electrode array that is inserted into the cochlea through an insertion sheath instead of a stylet, was released. Initial studies conducted with this electrode, designed for a perimodiolar position within the cochlea, demonstrated the feasibility of HP [13, 14]. However, it also became clear that this new, very flexible electrode carried a potential risk of tip fold-over during insertion [15] a fact that impedes HP.

The aim of this retrospective study was examine the degree of HP in a large group of patients implanted with the SM electrode carrier and evaluate if HP is reliably possible. This could have an impact on the clinical application of SM in patients with residual hearing.

## Method

### Study population

Ethical approval for this retrospective study was obtained from the local ethics committee (No 20-592). Records included 62 patients/75 ears implanted with a CI between January 2015 and March 2020. All of them received an SM electrode in the form of a C1532 or C1632 implant (Cochlear Ltd., Macquarie, Australia) at a university hospital otolaryngology department. For further analysis, it was necessary to exclude patients (n = 2); 4 ears in whom postoperative pure tone audiometry was not available.

Cases in which the electrode array required revision with another electrode carrier were also excluded from subsequent HP analysis. Revision surgery was required in 9% (7/75) of cases. The reasons were as follows: Six revisions were due to tip fold-over (8%). In one case (1%) the implant had to be replaced due to an electrode defect, probably caused during the insertion process. In six cases a different implant and electrode array was used for reimplantation, namely the *Contour* electrode. In one case the same SM electrode carrier was successfully reimplanted and data could be analysed.

A total of 51 adult patients (27 males, 24 females) resulting in 60 ears were finally included in the HP evaluation. Of these, 52 ears had received a *CI532* implant and 8 had received the later *CI632*. The mean age at implantation was 60.9 ± 15.2 years (range 17.5–89.7 years). All patients underwent routine preoperative cranial magnetic resonance imaging and fine slice computed tomography scans prior to the first implantation to exclude cochlear malformations or retrocochlear pathologies. Ten patients underwent bilateral cochlear implantation with SM electrode arrays on both sides. In six cases the second side was implanted sequentially and in two cases bilateral implantation was performed simultaneously. All patients had some measurable degree of residual hearing within the low frequencies, but audiometric thresholds did not necessarily meet the recommended indication criteria for EAS. Table 1 shows the causes of hearing loss for the 51 patients.

**Table 1:** Etiology of hearing loss

Etiology	No. of cases
Progressive sensorineural hearing loss	29
Result of one or more hearing loss events	11
Congenital	4
Genetic	3
Ototoxic	1
Cholesteatoma	1
Otosclerosis	1
Prior brain surgery	1

### Surgical procedure

In all cases included in this study, cochlear implantation was performed using a hearing and structure preservation technique [5, 16]. Before performing surgeries with the new electrode, all surgeons received training on handling the SM from the manufacturer. The round window was exposed by removing the promontory overhang. After placing the implant in the prepared skull bed, the round window was perforated with a needle. In cases, where the maximum diameter of the electrode sheath did not fit the round window, the hook region of the cochlea was reduced. The sheath with the loaded electrode array was inserted up to the silicone marker ring, using the facial nerve as a landmark for orientation, as suggested by the manufacturer. The array was then released through the sheath, which was carefully withdrawn after full insertion of the electrode. The second of the three white marker rings was preferably positioned in the area of the round window. There were slight variations depending on the size of the cochlea, and in some cases the third ring was reached. Over-insertion by active repositioning of the electrode-carrier was avoided. To complete the procedure, the opening of the cochlea around the electrode array was sealed with the patient’s temporal fascia. Finally, the electrode carrier was placed in the mastoid cavity without any obvious mechanical tension to avoid possible electrode migration [17]. Electrophysiological measurements were conducted intraoperatively. These included the assessment of stapedial reflexes for each electrode contact, the neural response telemetry and spread of excitation measurements. Our intraoperative monitoring did not include cochlear microphonics measurements. On the day of surgery or the first postoperative day, a cone beam computer tomography was conducted to ascertain the position of the electrode and to rule out the possibility of tip fold-over.

### Audiometric assessment

Perioperative and postoperative pure tone audiometry results were collected from 54 patients. Audiometric measurements were performed in sound-isolated rooms (IAC Acoustics, Winchester, UK) equipped with *CA 540/2* clinical audiometers (Hortmann AG, Neckartenzlingen, Germany) and *HDA 200* audiometric headphones (Sennheiser, Hannover, Germany). Calibration was performed regularly according to the manufacturers’ specifications.

The hearing threshold (air conduction) of the implanted side was recorded at the frequencies 125 Hz, 250 Hz, 500 Hz, 1 kHz, 2 kHz, 3 kHz, 4 kHz, 6 kHz and 8 kHz. In cases in which the air conduction threshold exceeded the technical measurement range of the audiometer, the maximum audiometer output level (Table 2) was utilised.

**Table 2:** Maximum audiometer output in dB for the frequencies in kHz

0.125	0.25	0.5	1	1.5	2	3	4	6	8	[kHz]
85	100	110	110	110	110	110	110	110	105	[dB]

Consequently, the maximum score for pure tone average (PTAmax) was set to 110 dB.

Frequency-specific pure-tone audiometry of residual hearing was performed at three time points:

- (T0) preoperatively.
- (T1) within 30 days postoperatively.
- (T2) later time point (20.7 ± 17.6 months) during follow-up.

**Fig. 1:**
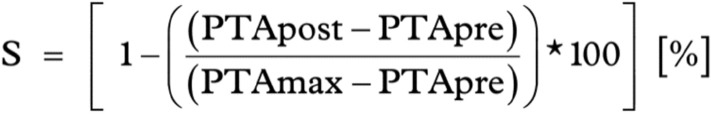
Formula for the hearing perservation classification system proposed by Skarzynski et al. [18]

In order to facilitate comparison of postoperative HP results with other studies, the pure-tone average in low frequencies (PTAlow) and the HEARRING group formula (Fig. 1) [18] were used. This equation makes the classification independent of initial hearing and eliminates the bias that worse preoperative hearing results in falsely better postoperative hearing outcomes. The calculated percentage can then be utilised to categorise the extent of HP as follows: over 75% equals a complete HP, between 75 and 25% partial HP, below 25% minimal HP and no measurable PTA results in a loss of hearing.

### Statistical analysis

Descriptive statistics were used to report demographic data.

The audiological data of this study were analysed, and charts were generated using Excel, Office 365 (Microsoft Corp, Redmond, Washington, USA). The statistic program GraphPad Prism Version 9 (GraphPad Software, Inc. San Diego) was used for statistical testing and generating graphic illustrations. The one-tailed t-test for unpaired and paired samples was calculated. The significance level for rejecting the null hypothesis was set at p < 0.05. A p-value of less than 0.05 was considered statistically significant.

## Results

### Audiometry

**Fig. 2:**
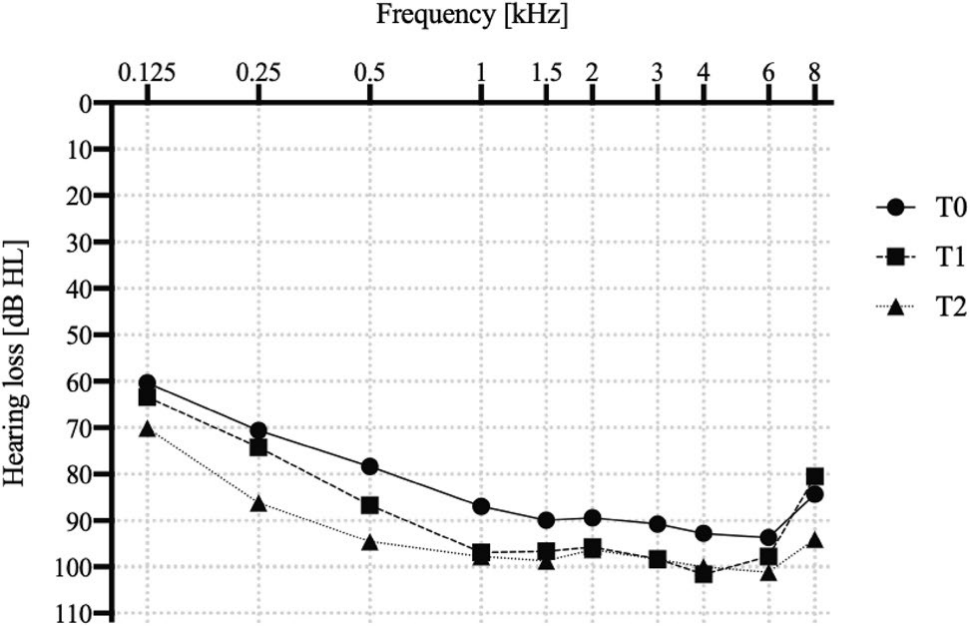
HP (mean values) at T0 = solid line with circles (n = 60), T1 = dashed line with squares (n = 39) and T2 = dotted line with triangulars (n = 48). With regard to low frequencies, no notable decrease was evident between T0 and T1. Notably, a statistically significant hearing loss was observed in higher frequencies. Between T1 and T2, a significant shift also occurred for low frequencies

Preoperative test results were available for all patients, resulting in 60 pure-tone audiometric data being recorded at time point T0 (preoperative). Over time, n = 39 data were collected at time point Tl (within the first 30 days postoperatively) and n = 48 data were collected at time point T2 (later time (20.7 ± 17.6 months)). The results for the patients at all three time points are shown in Table 3 and visualised in Fig. 2.

**Table 3:** Frequency-specific comparison of the mean differences (∆) in hearing [dB] at each measurement point; comparison of the amount of decrease in decibels and the corresponding p-values, significant p-values are shown in bold type

	Frequency (kHz)									
	0.125	0.25	0.5	1	1.5	2	3	4	6	8
Mean value T0 [dB]	60.3	70.6	78.3	86.9	89.9	89.4	90.8	92.8	93.7	84.3
Mean value T1 [dB]	63.4	74.2	86.7	96.9	96.7	95.8	98.3	101.6	97.8	80.5
Mean value T2 [dB]	70.1	86.1	94.6	97.8	98.8	96.3	98.4	100.0	101.1	94.1
*T0–T1*										
∆ [dB]	− 3.1	− 3.6	− 8.3	− 10.0	− 6.8	− 6.4	− 7.6	− 8.8	− 4.1	3.8
p-value	0.1920	0.1988	**0.0129**	**0.0005**	**0.0156**	**0.0304**	**0.0123**	**0.0027**	**0.0425**	0.1032
*T1–T2*										
∆ [dB]	− 6.7	− 11.9	− 7.9	− 0.9	− 2.1	− 0.5	− 0.1	1.6	− 3.4	− 13.5
p-value	**0.0157**	**0.0021**	**0.0135**	0.3733	**0.0009**	0.4392	0.4862	0.2706	**0.0293**	** < 0.000**
*T0–T2*										
∆ [dB]	− 9.8	− 15.6	− 16.3	− 10.9	− 8.8	− 6.8	− 7.7	− 7.3	− 7.5	− 9.7
p-value	**0.0012**	** < 0.000**	** < 0.000**	** < 0.000**	**0.0009**	**0.0118**	**0.0058**	**0.0047**	**0.0003**	** < 0.000**

Between T0 and Tl, there was no significant decrease in residual hearing for the frequencies 125 and 250 Hz. However, for frequencies higher than 250 Hz, there was a significant decrease of 6 dB on average (p < 0.05). In the period up to the next follow-up (T1–T2), residual hearing showed a significant deterioration of 11.9 dB at 250 Hz and 6.7 dB at 125 Hz (p < 0.05). When comparing the pre-operative test (T0) with our long-term results (T2), a significant deterioration can be observed at all frequencies (p < 0.05). The mean long-term deterioration was − 10 dB.

**Fig. 3:**
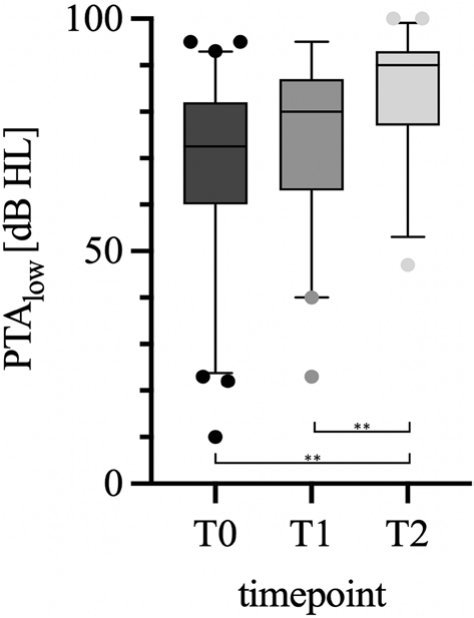
Box plot (median and quartile) of PTAlow at each measurement time point from black (T0) to light grey (T2), preoperatively (T0) n = 61, within 30 days postoperatively (T1) n = 39 and later time point (20.7 ± 17.6 months) during follow-up (T2) n = 48; *p < 0.05, **p < 0.005. A statistically significant reduction was observed for PTAlow between T0 and T2, as well as between T1 and T2

In addition, PTAlow (125–500 Hz) was calculated for a more detailed assessment (Fig. 3). There was no significant deterioration between the first two time points, but there was a significant hearing loss from T1 to T2, with an average of − 9.3 dB (p = 0.0025). Comparing T0 to T2, there was also a highly significant deterioration of − 13.9 dB on average (p < 0.000).

**Fig. 4:**
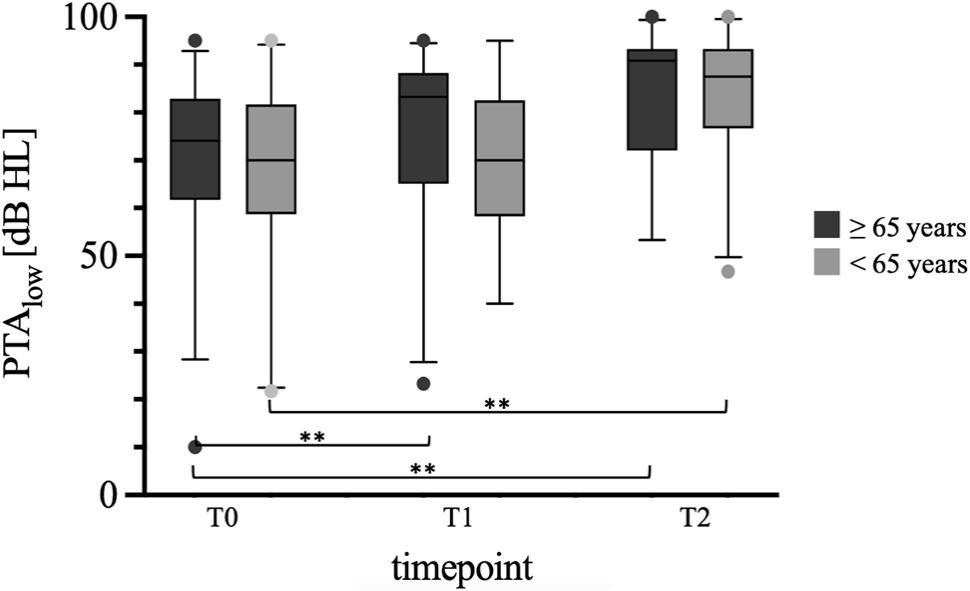
Comparison of PTA low at each measurement point divided into patients ≥ 65 years (dark grey on the left) and < 65 years (light grey on the right); **p < 0.05. A significant decline was observed in older patients between T0 and T1, whereas this was not the case in younger implantees. In both groups, a significant PTAlow deterioration was noted between T0 and T2. The p-values are presented in Table 4 for reference

To assess whether age was an influencing factor to progression of hearing loss, the patients were divided into two groups, younger (< 65 years; n = 32) and older (≥ 65 years; n = 28). There was no significant hearing loss in the younger group between T1 and T2, whereas there was significant hearing loss in the older cohort during this period. There was no significant deterioration in residual hearing between T0 and T1 in either group. When the different groups were compared individually at each time point, no significant deviation was found (Table 4 and Fig. 4).

**Table 4:** Comparison of the mean PTAlow at each measurement point divided into patients ≥ 65 years and < 65 years **p < 0.05

Age	n	Timepoint	PTAlow [dB]		p-value
	32	T0	70,94	T0-T1	0,1613
< 65 years	22	T1	77,12	T1-T2	0,1240
	26	T2	83,01	T0-T2	**0,0101**
	28	T0	68,39	T0-T1	0,3325
≥ 65 years	17	T1	70,59	T1-T2	**0,0026**
	22	T2	84,32	T0-T2	**0,0004**

In this context, bold numbers are used to indicate a significant score

### HEARRING classification

**Fig. 5:**
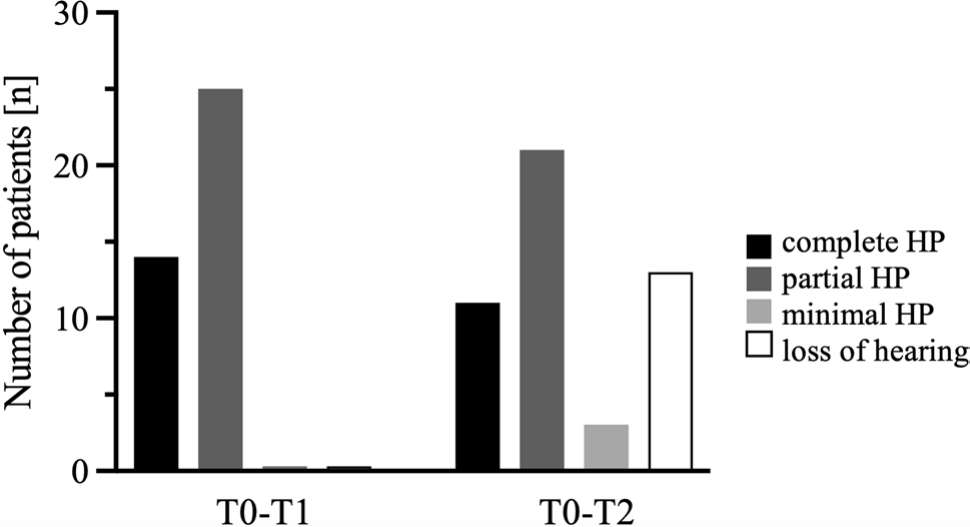
Number of patients [n] with complete, partial, minimal preservation of hearing (HP) and hearing loss. Timepoints were designated as preoperative (T0), up to 30 days postoperative (T1), and a later time point during follow-up (T2) (20.7 ± 17.6 months). The number of patients exhibiting complete, partial, or minimal preservation of hearing (HP) and hearing loss. Timepoints were designated as preoperative (T0), up to 30 days postoperative (T1), and a subsequent time point during follow-up (T2) with an average interval between measurements of 20.7 ± 17.6 months. The bars illustrate the number of patients categorised by their hearing preservation status and the change in this categorisation from the preoperative to the T1 and T2 measurements. It was evident at the later timepoint that patients with complete hearing preservation and those with partial hearing preservation exhibited a reduction, while cases with minimal hearing preservation and cases with total hearing loss increased

Using the HEARRING classification system [18], the average short-term (T0–T1; n = 39 HP score was 70%, which corresponds to partial HP. On an individual basis, 14 patients (36%) had complete HP and 17 (64%) had partial HP. No events of hearing loss were observed. In the long term (T0–T2) no measurable hearing could be observed in 13 cases (20%). For the remaining patients (n = 35), the average HP was 61%, which also still corresponds to partial HP. During this period, 11 patients (31%) had complete HP, 21 (60%) had partial HP and 3 (9%) had minimal HP (Fig. 5).

## Discussion

This study retrospectively examined HP rates in patients implanted with an SM electrode array. SM implantation resulted in an average partial HP of 70% within the first year, calculated using the HEARRING formula. This decreased to 61% at long-term follow-up, still resulting in partial HP. Pure tone audiometry showed no significant deterioration in low frequency hearing within the first year after surgery. In the long term, the results of this study showed a low frequency hearing loss of − 13.9 dB compared to previous thresholds. Across all frequencies, there was an average decrease of − 10 dB. There was evidence of accelerated hearing loss in older patients.

Given the recent preference for straight LW electrodes in HP surgery, it becomes pertinent to inquire whether residual hearing can be reliably preserved with pre-shaped designs that offer the distinct advantage of perimodiolar positioning. The use of LW arrays has been demonstrated by numerous studies to offer a favourable chance of hearing preservation. For example, Helbig et al. [16] conducted a longitudinal study examining the long-term hearing preservation potential of various LW electrode types in 103 ears. The results demonstrated that long-term HP was a viable option, although PTAlow exhibited a further decline, which was likely attributable to the varying rates of hearing loss progression among individuals. HP was classified as follows: complete HP for shifts of ≤ 10 dB, partial HP between 10 and 30 dB, and minimal HP for ≥ 30 dB. Complete postoperative hearing preservation was achieved in 25% (n = 52) of the study group, while partial preservation was observed in 60% of cases. The long-term results demonstrated partial HP in 53% of cases, with a mean follow-up period of 43.0 ± 18.3 months after surgery. The preoperative PTAlow exhibited a decline of − 11.7 dB over the course of 12 months, which is analogous to our findings of − 13.9 dB in the longer-term observation period. A complete loss of residual hearing was observed in 22 out of the 103 cases (21%), which is comparable to the 20% hearing loss events observed with the SM electrode carrier. Other hearing preservation rates reported in the literature for LW electrodes range from 54 to 88%, likely dependent on the length of the electrode and the classification used [19–22]. In contrast with these studies, Manjaly et al. [23] evaluated two types of LW electrodes of different lengths using the HEARRING formula, with an average follow-up period of 8 months in 52 ears. The results indicated that, following cochlear implantation, there was complete HP observed in 33% of cases, partial HP was seen in 42% of cases, and minimal or no HP was evident in 25% of cases. The mean level of HP in the cohort was 55.5%. These findings are comparable to our long-term results, wherein 31% of patients exhibited complete HP and an average score of 61%. Furthermore, the literature has demonstrated that HP is also feasible following surgery with long LW electrodes (≥ 28 mm) [﻿^24^], a length that is currently not available for perimodiolar electrodes. It can thus be concluded that the results of this study demonstrate comparable outcomes to those observed with the use of longer LW arrays in SM implantation.

Additionally, reports of favourable hearing preservation outcomes with SM electrodes are available in the literature [25–31]. Instead of utilising the HEARRING classification system, the majority of these studies describe HP in terms of PTA or PTAlow deterioration. For example, Ramos-Macías et al. [32] investigated HP in patients with an SM electrode and demonstrated that only one in ten patients with a *CI532* exhibited a complete loss of residual hearing at three months post-surgery. Notwithstanding, patients exhibited additional progressive hearing loss. After twelve months, 70% of the *CI532* cohort met the criteria for HP, defined as a hearing threshold reduction of 15 dB or less within the 125–750 Hz range. In comparison to the aforementioned results with *CI532*, all patients in the *CI512* control group who underwent implantation exhibited a complete loss of residual hearing. In a further study with comparable results, Lee et al. [33] observed complete or partial HP in 74.3% (n = 39) of cases one month after surgery with the SM electrode. After a twelve-month follow-up period, the prevalence of HP was observed to have decreased to 66.7% (n = 18). The minimum incidence of hearing preservation or loss of function was 25.7% within the first month (n = 39) and decreased over time to 33.4% at the twelve-month postoperative time point (n = 18). These findings are consistent with the hypothesis that surgery with the thin preformed electrode array can result in partial or even complete hearing preservation in the majority of cases. Iso-Mustajarvi et al. [28] demonstrated the most favourable outcomes with SM to date, resulting in complete HP in 41% and partial HP in 59% of cases using the HEARRING classification.

A comparison of the lateral wall and the SM perimodiolar design with regard to reliability for HP results appears to yield comparable outcomes. The observed decline in HP rates, irrespective of electrode type, indicates that other factors may be contributing to the hearing loss over time [25, 34].

The question of whether children exhibit superior HP rates following implantation, as reported by Zanetti et al. [35], or whether no significant impact is observed, as suggested by other reviews, remains a topic of debate [36]. In the adult population presented here, we observed minor discrepancies in the deterioration dynamics between the younger (< 65 years) and older groups. However, no statistically significant differences were identified in the final average outcome. It seems highly probable that progressive hearing loss is more prevalent in older patients, which would explain the further deterioration observed in this study. Extending this reasoning to its logical conclusion, it can be deduced that neither the electrode design nor the surgical trauma itself exerts a significant influence on the hearing outcome following implantation. The only exception to this would be the cases of tip fold-over, which have been documented in the literature for this type of electrode, and which also occurred in 8% of cases in this study.

### Study limitations

A total of 13 cases (20%) of complete hearing loss were observed in the present study. As patients (n = 5) with no audiometric data available after surgery were excluded from this study, it is possible that potential hearing loss may have been the reason for not taking further measurements. Furthermore, it should be acknowledged that six patients who were initially scheduled for SM underwent a revision procedure with a different array and were therefore excluded from the study. It can be concluded that these cases should be added to the group of patients with complete hearing loss. It can be reasonably assumed, therefore, that the actual incidence of hearing loss events is higher.

As the study was retrospective in nature, the results for hearing thresholds immediately after implantation were not consistently available. It is therefore not possible to determine with certainty whether this deterioration was due to direct insertion trauma or a longer-term effect, such as a postoperative immune response within the cochlea.

The study only included results from patients who had an SM electrode carrier implanted. Patients with anatomical variations, such as a narrow chorda tympani angle, which required the use of alternative electrodes intraoperatively, were not included. It is therefore possible that the results would have shown a greater reduction in residual hearing if all candidates selected for SM implantation had been included.

The study does not allow for the resolution of the fact that residual hearing decreases first in the higher frequencies and then in the lower frequencies. The hearing loss may have occurred either because of individual threshold shifts or as a result of trauma to the basal cochlear structures during insertion. The length of the SM electrode does not allow for a deep insertion into the apical region of the cochlea, which may help to preserve low frequency hearing.

We acknowledge the wide range of follow-up times at the later time point (T2), with a mean of 20.7 ± 17.6 months postoperatively. This variability is inherent to the retrospective nature of our study and reflects the heterogeneity in clinical follow-up schedules between patients. To maximise the robustness of our analysis and avoid the introduction of exclusion bias, we decided to include all patients with measurable hearing outcomes at the later time point (T2), even if the time intervals varied. Excluding patients with shorter follow-up times would have reduced the sample size and potentially introduced selection bias. One patient was assessed less than two months after surgery, while all other patients were assessed at least three months after surgery. In addition, 38 of the 60 patients were assessed more than six months after surgery, demonstrating that the majority of patients were indeed followed for a longer period of time.

## Conclusion

The results demonstrate that residual hearing preservation is possible after cochlear implantation with the SM electrode carrier, particularly at low frequencies. It is important to consider the incidence of hearing loss due to tip fold-over during insertion when counselling patients with residual hearing. The SM electrode carrier offers a comparable chance of HP to LW electrodes, though it should be noted that the LW arrays allow a deeper insertion. The results of this study indicate that SM may be a suitable option for patients with residual hearing. However, further data and larger groups are required to assess the long-term efficacy of SM in patients considered for HP.

## Data Availability

The data that support the findings of this study are available from the corresponding author upon reasonable request.
